# Advanced surgical tool: Progress in clinical application of intelligent surgical robot

**DOI:** 10.1002/SMMD.20220021

**Published:** 2022-12-27

**Authors:** Chao Li, Tongtong Zhang, Haoran Wang, Zhiyong Hou, Yingze Zhang, Wei Chen

**Affiliations:** ^1^ Department of Orthopaedics the Third Hospital of Hebei Medical University Orthopaedic Research Institution of Hebei Province NHC Key Laboratory of Intelligent Orthopaedic Equipment Shijiazhuang China

**Keywords:** clinical application, computer‐assisted, surgical robot, surgical tool

## Abstract

Surgical robot is a revolutionary tool conceived in the progress of clinical medicine, computer science, microelectronics and biomechanics. It provides the surgeon with clearer views and more comfortable surgical postures. With the assistance of computer navigation during delicate operations, it can further shorten the patient recovery time via reducing intraoperative bleeding, the risk of infection and the amount of anesthesia needed. As a comprehensive surgical revolution, surgical robot technique has a wide range of applications in related fields. This paper reviews the development status and operation principles of these surgical robots. At the same time, we also describe their up‐to‐date applications in different specialties and discusses the prospects and challenges of surgical robots in the medical area.

## Graphical abstract

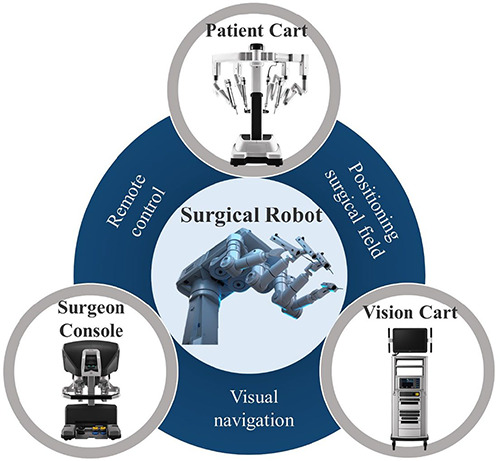

1

**Key points**

- Presenting a comprehensive discuss of the technical principles of surgical robots and research progress of work field.
- Highlighting the applications and challenges of surgical robots for different disciplines.
- Summarizing the technical features and operating procedures of surgical robots.
- Discussing the prospect and future direction of surgical robots in clinical application.

## 2 INTRODUCTION

Introducing the concept of robots into the surgery field has always been a wonderful vision for clinical medicine. In last decades, surgery has been successfully integrated with different types of machines, which are gradually perfected as sophisticated operating systems for artificial intelligence surgery. Generally, the purpose of surgical robots is merely assisting surgeons to perform surgery better rather than replacing them, thus the deep learning capabilities are not indispensable in the beginning. However, faced with the growing risks due to the high intensity of modern surgery, surgeons generated increasing urgent and diversified needs for these surgery machines. As the product of this demand, surgical robot has been recognized by lots of clinical and scientific researchers as a representative of high‐end intelligent medical devices. In 1985, Kwoh completed the world's first robotic combined surgery successfully for neurosurgical biopsy in the United States.^1^ However, the used industrial robot did not work well with the surgeon. Later, with the continuous development of laparoscopic technology, especially the emerged endoscopic systems, the next generation surgical robot “Zeus” was equipped with an endoscope and can perform minimally invasive surgery remotely.^2^ The Zeus system was inspired by the dexterity of the surgeon's arm and hand, and its mimicry of the human arm gave it a more bionic mode of operation. In 1999, the “da Vinci” surgical robot, representing the highest level of technology at that time, was introduced and approved by the Food and Drug Administration (FDA) for use in human abdominal surgery the following year.^3^ With a spider‐like metal exterior, the da Vinci not only accomplishes naked‐eye 3D imaging but also eliminates hand tremors, allowing surgeons to operate consistently. Up to March 2020, the da Vinci surgical robot had participated in more than 7.2 million surgeries of all types. As the demand for surgical robots from medical units and patients is increasing, the global surgical robot field holds tremendous energy and is about to induce a new round of development.

**FIGURE 1:**
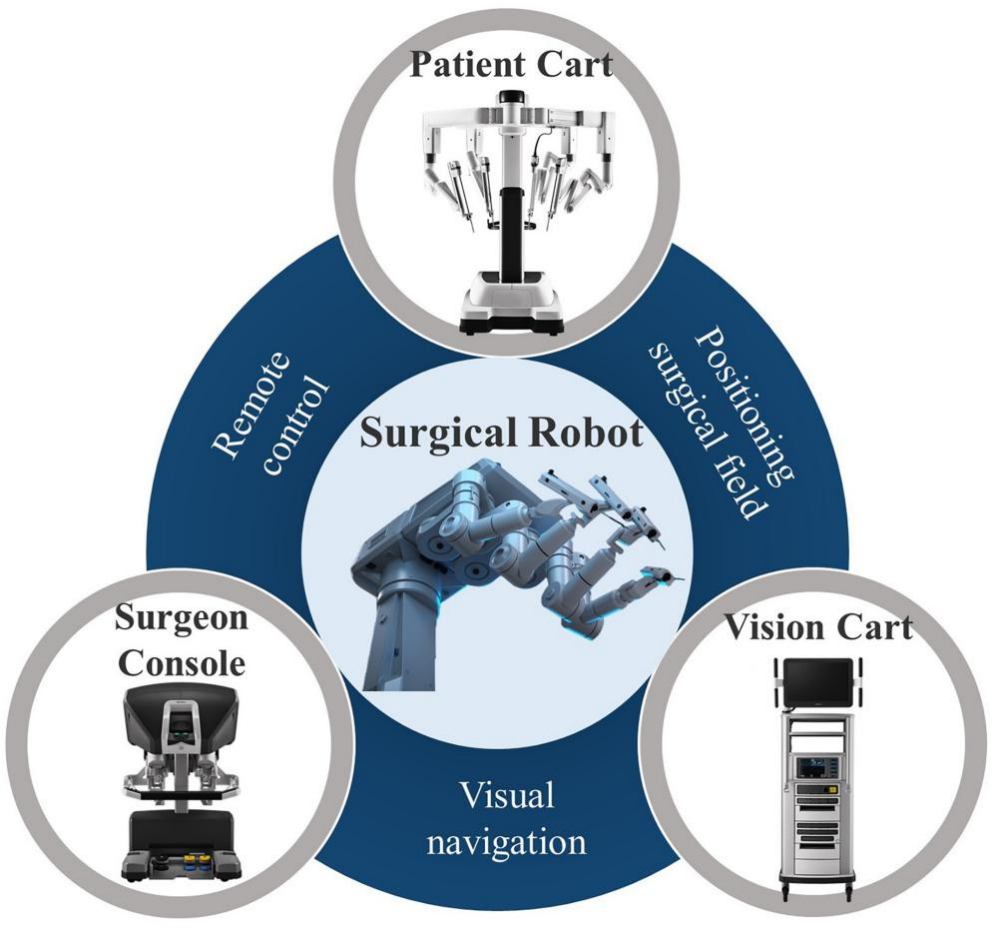
Schematic diagram of the basic components of a surgical robot. Reproduced with permission.^6^ Copyright 2022, Intuitive Surgical.

Current surgical robots are mostly designed to assist surgeons rather than to obtain a fully automated and autonomous operation.^4^ However, it is the structure of the surgical robot that gives it a “synergistic” working principle. Mainstream surgical robots consist of a main console, a robotic arm system, and an optical imaging system (Figure 1).^5^, ^6^ Surgeons usually perform the entire operation in a seated position in front of a console to overcome the fatigue and muscle strain associated with longer operating times. Facing numerous joysticks, pedals, or controllers, surgeons can remotely control robotic assistive devices, such as robotic arms, by wired or wireless means, without touching the patients.^7^ As the core component of surgical robots, the surgical robotic arms are connected to the console, which also direct contact with the patients. It is crucial that the robotic arm operates as expected. Usually, the robotic arm will have many sub‐arms corresponding to the functions of suction, grasping, probing, or electrocoagulation.^8^ The imaging system is designed to transmit images of the operative field to the surgeon in front of the console, allowing the attending surgeon to have a clearer identification of the current anatomical position.^9^ The probe attached to the imaging table also has a magnification function compared to direct visualization, which helps to increase the precision of the procedure.

Surgical robots are changing the traditional concept of surgical operation. From the perspective of operators, the core purpose of surgical robots is assisting surgery for a better completion via their digital and mechanized functions. The presence of console could allow the operator with a more comfortable sitting position for reducing fatigue and enhancing concentration.^10^ Moreover, advanced surgical robots have the ability to eliminate hand tremors, which is a strong corrective effect for procedures requiring high precision.^11^ Besides, the operator–patient separation mode combined with the aggregation function of multiple robotic arms allows the surgery to be completed by fewer operators. From the perspective of patients, smaller incisions, less blood loss, and shorter recovery time of robot‐assisted surgery are also very attractive.^12^ Table 1 presents a comparison of surgical robots with conventional surgery.

**TABLE 1:** Comparison of surgical robot and traditional surgery

Surgery methods	Posture	Auxiliary effect	Limit of people	Visual effect	Incision size	Recuperation time
Traditional surgery	Usually standing	None	Multiple people	Normal view	Large	Long
Surgical robot	Sitting	Anti‐hand tremor	Single people	Enlarge view	Small	Short

In recent years, rapid developments in mechanical engineering, artificial intelligence, and medicine greatly facilitated the possibility of using surgical robots in clinical disciplines such as orthopedics, urology, general surgery, and obstetrics and gynecology.^13^ As the most powerful tools for clinical surgery, surgical robots are easily adapted and matched for various types of surgery owing to the powerful human–machine cooperation ability and smooth, humanized operation sense.^14^ In this paper, we reviewed the clinical application directions and research status on surgical robots. Besides, we highlighted and discussed the current operation mode of surgical robots, and then summarized the technical characteristics and the direction of their subsequent development.

## 3 STATUS OF RESEARCH ON CLASSIFICATION OF SURGICAL ROBOTS

The clinical application of surgical robots started a new chapter of intelligent medicine history. For surgeons, surgical robots cannot completely replace the primary surgical position, but they really benefit for minimal invasion and precise surgical requirements.^15^ Due to the multiple functions that guide surgical navigation or precise operations, the performance of surgical robots in different disciplines has finally convinced surgeons and patients (Table 2).

**TABLE 2:** Representative surgical robots

Name	Country/Company	Features	Applications	Literature
ROBODOC (TSolution‐One®)	Curexo technology, USA	The TSolution‐One® allows intraoperative placement of the prosthesis and precise osteotomy based on preoperative	Orthopedics	Liow et al.,^32^ 2017
VELYS™	DePuy Synthes, USA	No reliance on preoperative imaging and the arm can be placed directly at the head of the surgical bed	Orthopedics	Doan et al.,^38^ 2022
Cori Surgical System	Smith and Nephew, UK	Individualized implantation for each patient based on different parameters using visual cutting technology	Orthopedics	Smith + Nephew,^39^ 2022
SpineAssist®	Mazor Robotics, Israel	SpineAssist® automatically controls its robot arm according to a preset trajectory for screw insertion, etc.	Orthopedics	Lieberman et al.,^44^ 2006
MAZOR X STEALTH EDITION^TM^	Medtronic, USA	The MAZOR X STEALTH EDITIONTM arm contains several optical cameras that rotate 360° to assess the surgical surroundings, avoid collisions, and complete field scans	Orthopedics	Khan et al.,^51^ 2019
ROSA® Spine robot	Zimmer Biomet Robotics, USA	The robotic arm with tracking capability directed by a navigation system allows for minimally invasive circumferential fusion	Orthopedics	Lefranc et al.,^56^ 2016
da Vinci® Surgical System	Intuitive Surgical, Sunnyvale, USA	da Vinci® Surgical System is more like an advanced laparoscopic system that performs complex procedures through a minimally invasive approach	General Surgery	Tsuda et al.,^62^ 2015
Senhance® robotic platform	Asensus Surgical, USA	Senhance® robotic platform uses a new dynamic eye tracking system that focuses the camera on the field of view the surgeon is looking at	General Surgery	Samalavicius et al.,^78^ 2019
Versius® surgical system	CMR Surgical, UK	Not only optimized human–machine interaction system, but also has a V‐shaped wrist joint robot arm, easy to adjust while also easy to transfer and move	General Surgery	Haig et al.,^86^ 2019
TELELAP ALF‐X surgical system	SOFAR S.p.A., Italy	An open console is used, equipped with both an eye tracking system camera and a robotic arm	Gynecology	Fanfani et al.,^94^ 2016
NeuroArm	IMRIS‐deerfield, USA	The world's first MRI‐compatible image‐guided neurosurgery robot	Neurosurgery	Sutherland et al.,^101^ 2013
Anovo™ Surgical System	Momentis Innovative Surgery, USA	As the only FDA‐authorized transvaginal surgical robot, it is the surgical robot that penetrates the farthest into the body	Gynecology	Momentis Innovative Surgery,^98^ 2022
Aquabeam® system	PROCEPT BioRobotic, USA	The anatomical structures related to sexual and urinary function can be observed and preserved and the diseased tissue can be precisely removed	Urology	Misrai et al.,^90^ 2019

Compared to other surgical procedures, orthopedic surgery has numerous types of consumables, and usually strictly follows several biomechanical standards.^16^ Due to the diversity of orthopedic surgeries, the design of robots should consider different demands from surgeons. The common orthopedic surgeries are mainly divided into trauma surgery,^17^ spine surgery,^18^ and joint surgery.^19^ More than that, different types of surgery also correspond to specific surgical operations. For example, in the field of trauma, the most common operation is fixation repair of limb bone fractures like pelvis acetabulum, femur, and tibia.^20^, ^21^, ^22^ In common internal fixation of femoral neck fractures with hollow nails,^23^ internal fixation of femoral intramedullary nails^24^ and internal fixation of forearm double fractures,^25^ not only the type of plates and screws are necessary elements for successful surgery, but their placement is also an important influencing factor to ensure patient safety and better outcome. Compared to conventional naked eye localization, robotic localization is able to overcome the drawbacks of repeated use of X‐imaging fluoroscopy and 2D field of view.^26^ Precise positioning not only reduces secondary damage to soft tissues or bone, but also further reduces the incision and accelerates healing on a minimally invasive basis. However, fracture resetting robots are difficult to develop and there are still no clinically available products.

### 3.1 Joint surgery robot

In the joint surgery field, one of the most difficult procedures is hip or knee arthroplasty, which is also very common.^27^ If joint replacement procedures fail to achieve a good alignment, the issues of postoperative complications will occur. Although there are lots of achievements in arthroplasty in the last decade, the complications still cannot be ignored, such as prosthesis loosening, periprosthetic fractures or stress instability.^28^ Undoubtedly, the advent of surgical robots has given opportunities to overcome these common joint replacement problems.

**FIGURE 2:**
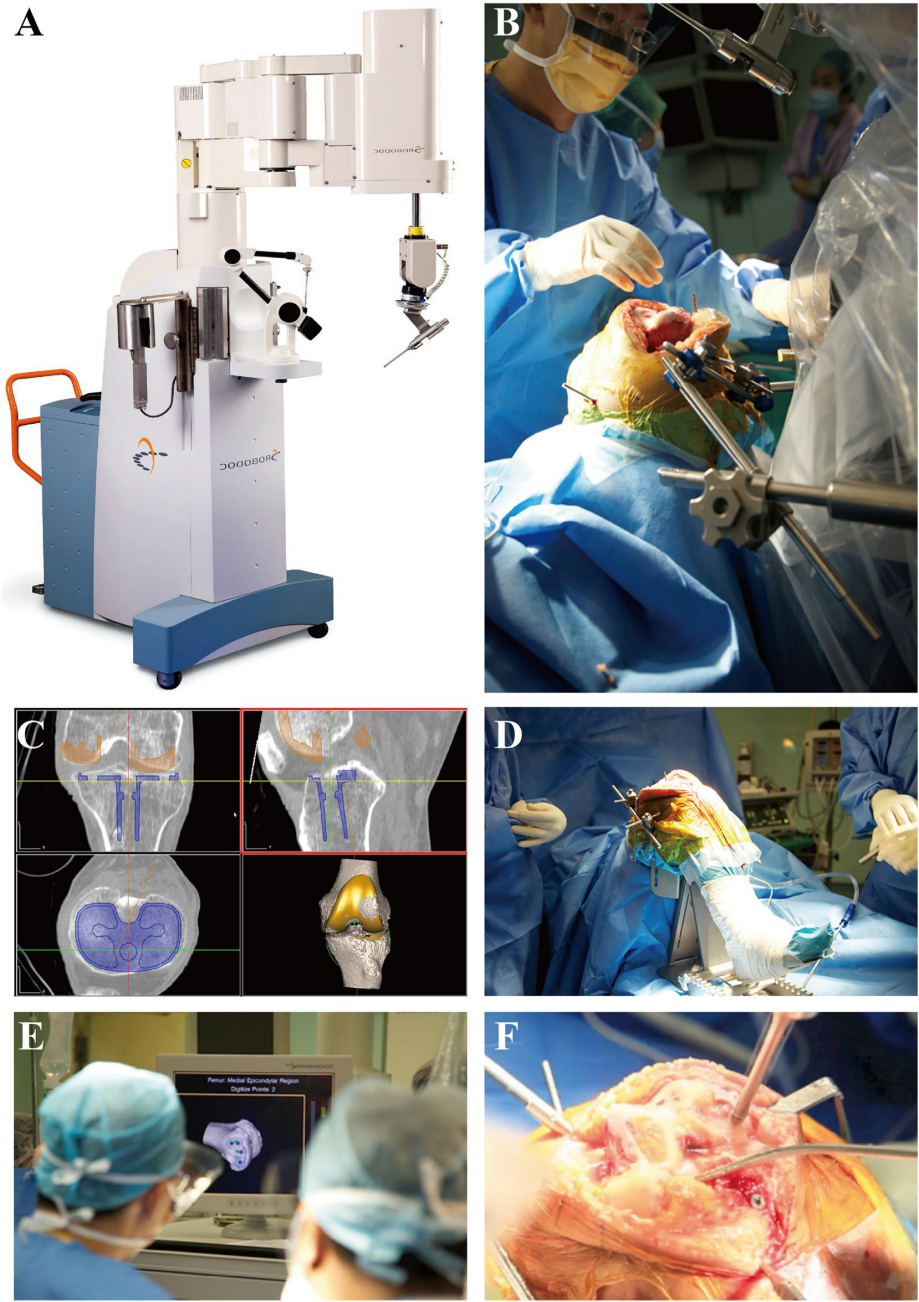
TSolution‐One® surgical robot. (A) Image of TSolution‐One® system. (B) TSolution‐One® with the surgeon. (C) Preoperative virtual surgery. (D) Personalized brackets. (E) Femoral imaging markers. (F) Robotic cutting of the femur. Reproduced under terms of the CC‐BY license.^32^ Copyright 2017, The Authors, published by EDP Sciences.

The commercially available orthopedic surgical robot, ROBODOC® (Curexo Technology Co.), was firstly reported in 1986,^29^, ^30^ then approved for orthopedic total hip arthroplasty (THA) in 1992.^31^ Subsequently, in 2014, the ROBODOC® system was upgraded and renamed as TSolution‐One® (Figure 2A). The TSolution‐One® is a fully automated surgical robot that can perform intraoperative prosthesis placement and precise osteotomy according to preoperative imaging data in a pre‐programmed manner^32^ (Figure 2B–F). The surgeon just needs to input X‐ray or CT data and the TSolution‐One® robotic arm could automatically place the prosthesis in the proper position accordingly and realize osteotomizing and irrigating with submillimeter precision simultaneously. In addition, it has a built‐in movement protection program that suspends operation and prompts an alarm when the incision or the robot arm itself is unprogrammed to shift. The TSolution‐One® could minimize human error while maintaining a relatively appropriate bone temperature. It not only allows fine cutting and placement but also reduces the risk of the procedure. After comparing the advantages and disadvantages of TSolution‐One® versus conventional surgery in terms of prosthesis placement, force line recovery, and recovery, Kim et al. reported that all patients in the TSolution‐One® group had better force line recovery than the conventional surgery group, despite the increased cost of the procedure.^33^ Nevertheless, some evidences still suggest that TSolution‐One® does not have a significant effect on postoperative knee function improvement compared to manual replacement.^34^, ^35^

**FIGURE 3:**
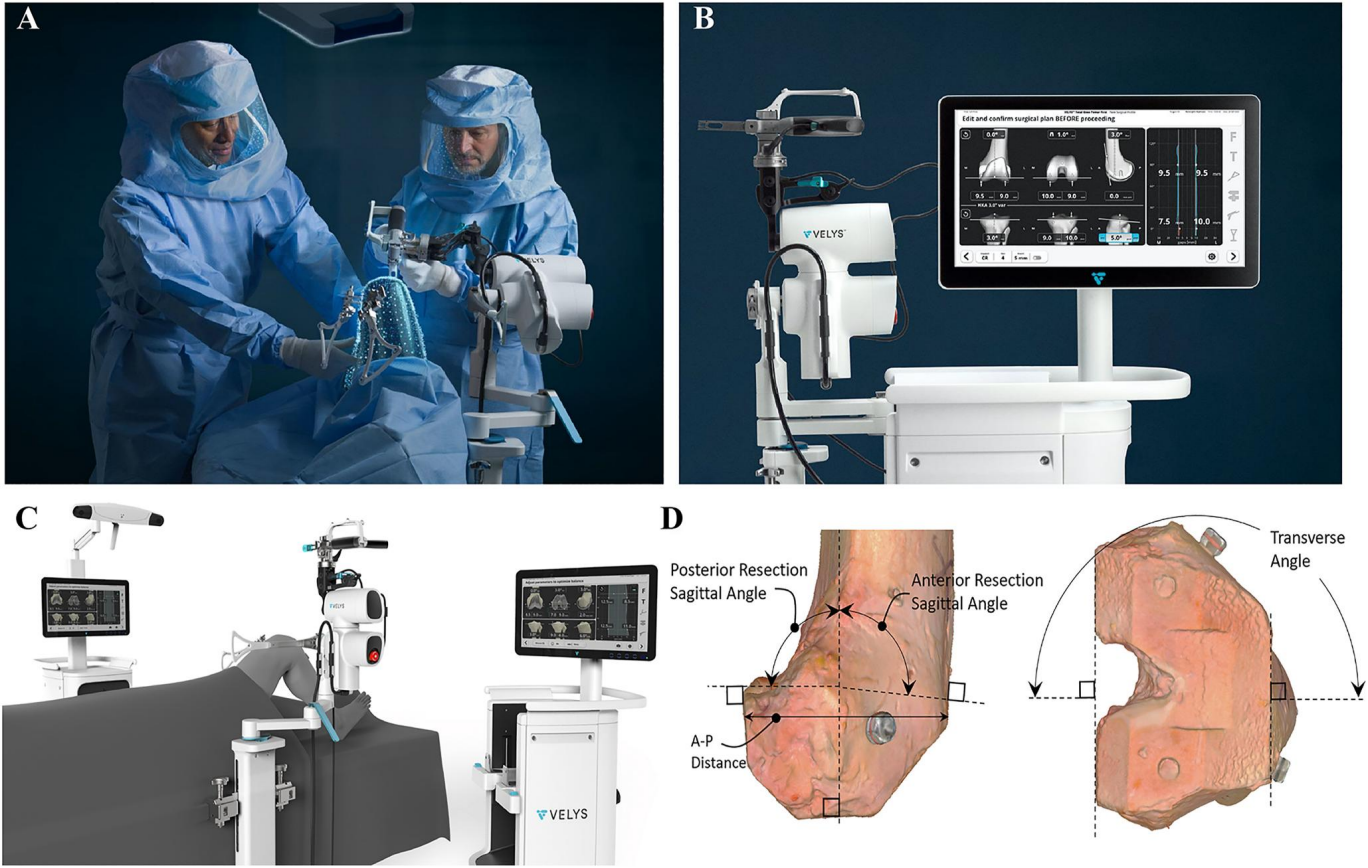
(A) VELYS™ is used in total knee arthroplasty. (B) Software systems for evaluation and forecasting. Reproduced with permission.^36^ Copyright 2022, DePuy Synthes. (C) VELYS™ includes an optical tracking system, a positioning needle and a robotic arm that is positioned at the head of the surgical bed. (D) Resection models produced by CT in the sagittal and cross‐sectional planes of the femur. Reproduced under terms of the CC‐BY license.^38^ Copyright 2022, The Authors, published by Elsevier.

Another VELYS™ (DePuy Synthes Co.) surgical robot was also approved by the FDA in 2021 for joint replacement (Figure 3A). It simplifies the process of joint replacement and optimizes the surgical protocol. Its compact size makes it easy to adapt to any operating room, freeing up more space for surgery. The VELYS™ arm can be placed directly at the head of the surgical bed, and its multifunctional and integrated structure can meet the different needs of surgery.^36^ Instead of relying on preoperative imaging, VELYS^TM^ could evaluate the degree of resection and forecast joint stability using joint space, bone volume, etc.^37^ The VELYS™ also automatically adjusts parameters to help the surgeon align the appropriate soft tissues through a self‐contained system (Figure 3B). The adaptive tracking technology high‐speed camera is equipped to allow VELYS™ to quickly identify the relative positions between the surgical instruments, the attending surgeon, and the patient during the operation. Doan et al. reported a cadaveric study of total knee arthroplasty using VELYS™ and conventional instruments.^38^ Based on preoperative and postoperative computed tomography data, the robot‐assisted replacement possessed smaller resection errors in different orientations (Figure 3C,D). Compared to traditional total knee arthroplasty, the VELYS™ improves the precision of the prosthetic anastomosis and reduces the incidence of postoperative complications.

**FIGURE 4:**
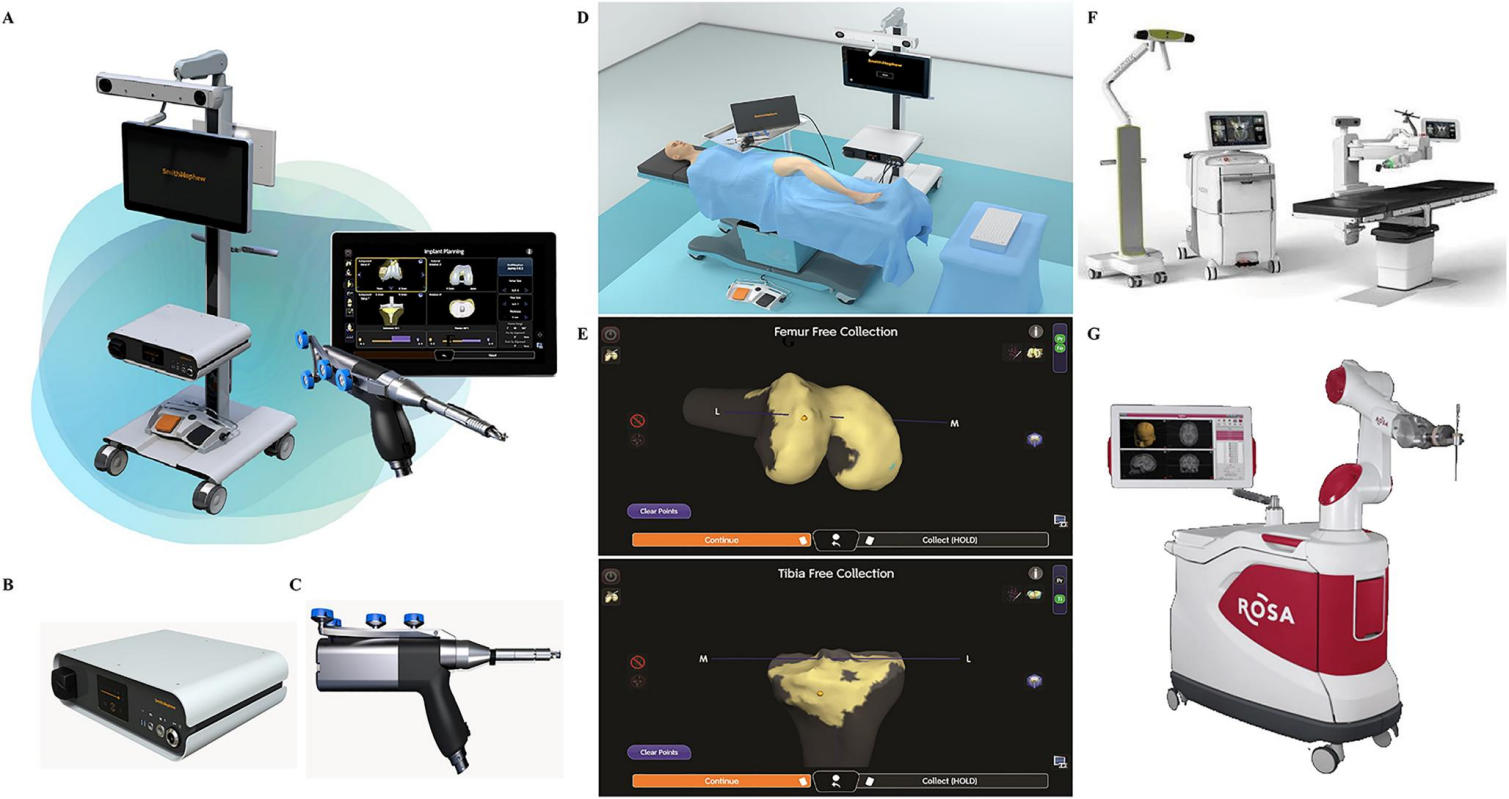
(A) Cori Surgical System. (B) Console. (C) Handheld mechanical handle. (D) Cori Surgical System in total knee arthroplasty. (E) Real‐time characterization of bone and cartilage through landmark collection without preoperative imaging. Reproduced with permission.^39^ Copyright 2022, Smith + Nephew. (F) Image of MAZOR X STEALTH EDITION^TM^. Reproduced with permission.^50^ Copyright 2017, Medtronic. (G) Image of ROSA® Spine robot. Reproduced with permission.^55^ Copyright 2017, ZIMMER BIOMET.

The Cori Surgical System (Smith and Nephew Co.) is a new generation of joint replacement surgery robots with integrated software, intelligent operating system, and data analysis system. The Cori Surgical System is an advanced handheld surgical robot that uses visual cutting technology to develop a personalized implantation plan for each patient based on different parameters.^39^ It has a high refresh rate tracking system, a real‐time imaging monitor, a master console, and a handheld mechanical handle (Figure 4A–C). CT or MRI data are not necessary for calibration and evaluation (Figure 4D,E). Its compact mechanical handle reduces unnecessary movement and cumbersome switching. The Cori Surgical System has great potential for use not only in knee arthroplasty but also in hip arthroplasty. Tim Parratt, one of the first surgeons in the UK to use the robot, praised the portability of the Cori Surgical System.^40^ At the same time, joint replacements assisted by the Cori Surgical System are less likely to be painful and have a lower revision rate.

### 3.2 Spine surgery robot

As a key component of the locomotor system, spine surgery is often regarded as a more difficult operation compared to the conventional extremity bone surgery because the spine is anatomically connected to numerous nerves and blood vessels.^41^ The introduction of robotics not only greatly improves the accuracy of surgery and reduces human errors but also shortens the learning curve and growth cycle of the surgeon. At the same time, the reduction of intraoperative radiation dose has also made spinal surgery robots increasingly popular and preferred by the majority of operators.^42^

The SpineAssist® (Mazor Robotics Co.), the first spine surgery robot that received FDA clinical use approval, is still in use on spine surgical tables around the world.^43^ Compared to conventional computer‐assisted navigation, the SpineAssist® is characterized by its ability to automatically control its robotic arm for screw placement based on a preset trajectory.^44^ In spinal fusion, the SpineAssist® calculates the optimal screw alignment coordinates based on preoperative imaging data.^45^ Following frame fixation and image acquisition, the pairing is finished, revealing the precise location on the patient.^46^ Sukovich et al. collected information on 14 completed spinal fusion cases and found that 93% of the procedures achieved the desired effect with the aid of SpineAssist®. Ninety‐six percent of the actual pedicle screw placement errors were less than 1 mm.^47^ Peng et al. performed a randomized controlled trial of 540 patients and 2476 pedicle screws, which showed a significant increase in screw placement accuracy and shorter irradiation time with robotic assistance compared to conventional surgery, but a significant increase in operative time.^48^

The MAZOR X STEALTH EDITIONTM (Medtronic Co.) has a detachable robotic arm and a computer‐like workstation^49^, ^50^ (Figure 4F). The MAZOR X STEALTH EDITIONTM is the latest version of the spine surgery robot that enables highly precise, predictable, and fully visualized surgical objectives through AI digitization and computerized navigation, significantly reducing intraoperative risks. Unlike the previous models, the MAZOR X STEALTH EDITIONTM arm contains several optical cameras that can be rotated 360° to assess the surgical surroundings, avoid collisions, and complete field scans.^51^ O'Connor et al. used the MAZOR X STEALTH EDITIONTM to place 90 pedicle screws after postoperative CT results showed 100% accuracy and not a single complication.^52^ Robot‐assisted screw placement maximizes accuracy and safety.^53^ In addition, MAZOR X STEALTH EDITIONTM has great potential to improve patients' prognosis. Good et al. conducted a single‐center retrospective study comparing the complication rates of conventional surgery with MAZOR X STEALTH EDITIONTM‐assisted surgery.^54^ All posterior spinal fusions assisted by the MAZOR X STEALTH EDITIONTM robot were performed without breakage in screw placement and with few complications and a low return rate, which can prove to be a reliable method of internal fixation.

The ROSA® Spine robot (Zimmer Biomet Robotics Co.) is a next‐generation spine robot that allows high‐precision intraoperative pedicle screw placement in conjunction with CT^55^ (Figure 4G). Its robotic arm with tracking capability can be directed by the navigation system to perform minimally invasive annular fusion.^56^ Due to the limitations of the navigation system, if the patient's reference shifts, all subsequent operations will be ceased. Lefranc et al. evaluated the accuracy of the ROSA® Spine robot for CT‐guided screw placement and showed that it has an accuracy range of 2 mm, which not only increases the mechanical advantage of pedicle screw fixation but also reduces the risk of toxicity after screw placement.^57^ The precise and complex procedure forces surgeons to have a longer learning curve and the cost of maintaining the robot increases. However, the ROSA® Spine robot can reduce patient length of stay and infection rates. Overall, the ROSA® Spine robot still has a high clinical application value.

### 3.3 General surgery robot

In general surgery, robots are widely accepted in various fields from hernia surgery to cholecystectomy or from strangulated bowel obstruction release to radical colorectal cancer surgery.^58^ With the widespread use of surgical robots in general surgery, the advantages of robot‐assisted surgery are increasingly recognized. Traditional gastrointestinal or hepatobiliary surgery is long, with large incisions, and is a great test of the surgeon's strength and the patient's recovery. Modern general surgery is moving in the direction of minimally invasive surgery and the use of laparoscopy is becoming more common. The advent of laparoscopy can reduce the length of stay of patients and reduce the incision and scarring.^59^ However, to some extent, laparoscopy also limits the flexibility of the surgeon and the interaction with the surgical operation.^60^ The hand‐to‐organ contact response is often irreplaceable when encountering unexpected intraoperative conditions such as bleeding and adhesions. Therefore, the use of surgical robots could compensate for the limitations of laparoscopy without losing the advantages of minimally invasive surgery.

**FIGURE 5:**
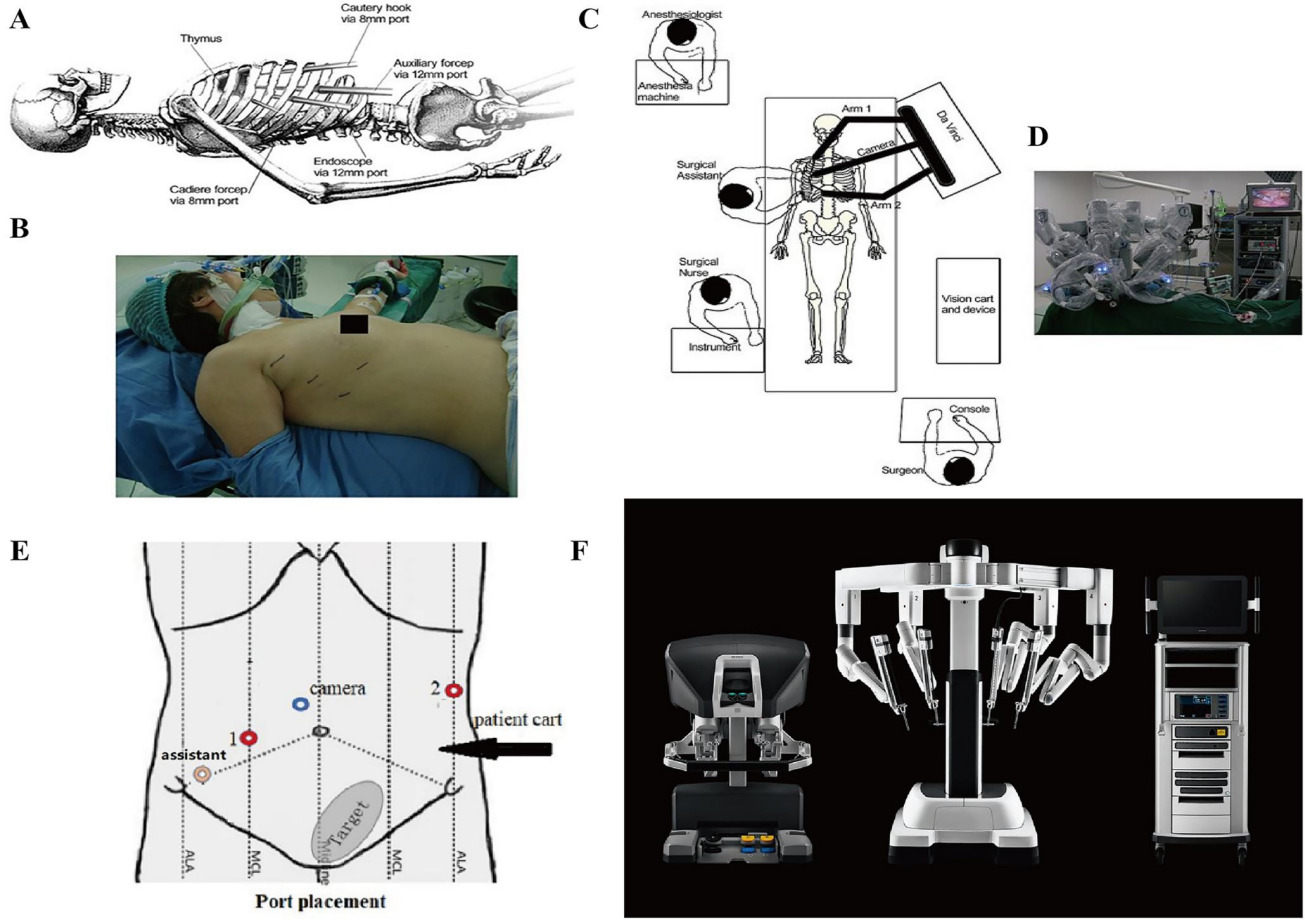
da Vinci® robot‐assisted thymectomy. (A) Schematic diagram of the position of the robot arm. (B) Schematic diagram of the position of the real patient. (C) Station of physician, anesthesiologist, and nurse. (D) Realistic operating room. Reproduced with permission.^69^ Copyright 2021, Wiley. (E) R‐type total rectal mesenteric excision (TME) port location: The camera port is located 2–3 cm near the upper umbilicus. Port 1 is located on the right midclavicular line (MCL), 15° below the umbilical level. The distance between port 1 and the camera port is at least 8–10 cm. Port 2 is located on the anterior axillary line, 15° above the umbilical level. The auxiliary port is located on the right MCL, 8–10 cm from port 1 and the camera port. Reproduced with permission.^77^ Copyright 2014, Wiley. (F) da Vinci® Surgical System. Reproduced with permission.^6^ Copyright 2022, Intuitive Surgical.

The da Vinci® Surgical System (Intuitive Surgical Co.) is a landmark invention as the benchmark for general surgery robots. As of June 2022, the da Vinci® Surgical System had a cumulative installed base of 7135 units worldwide. In 2022 Q2, the quarterly installed base reached 279 units.^61^ The da Vinci® Surgical System, jointly developed by MIT and IBM mainly, is currently the most advanced surgical robot. The FDA approved the da Vinci® Surgical System for a variety of clinical scenarios such as general surgery, cardiothoracic surgery, urology, and obstetrics and gynecology.^62^ In short, the da Vinci® Surgical System is more like an advanced laparoscopic system that performs complex surgeries through a minimally invasive approach. Data showed that surgical robots such as the da Vinci® Surgical System gradually took over the previous areas of traditional surgeries. Currently, there is a significant increasing use of the da Vinci® Surgical System in procedures such as inguinal hernia repair, cholecystectomy, and colectomy.^63^ During surgery by da Vinci® Surgical System, the surgeon only need to sit on the side and manipulate the arm via a controller rather than standing by the patients. Figure 5A–D shows the positioning of various people in the operating room during da Vinci® Surgical System‐assisted surgery. Although away from patients, da Vinci® Surgical System could in real time monitor what is happening in vivo through image transfer into a series of procedural actions. These technologies ensure a good sense of control and spatial distance for the surgeon. The da Vinci® Surgical System's robotic arm has multiple joints that allow flexibility in all directions to perform operations, such as cutting, grasping, suturing, or electrocoagulation, and can even perform actions that cannot be performed manually.^64^ This multi‐axis rotating robotic arm ensures a large field of view in the natural cavity, avoiding the reverse operation of a regular laparoscope and greatly improving the speed and accuracy of the surgeon.^65^ In contrast, the endoscopic 3D imaging lens of the da Vinci® Surgical System has a magnification effect that provides the primary surgeon with high‐definition stereoscopic images, thus allowing the surgeon to view the surgical area and anatomy more closely. High‐resolution images, remote synchronization, tremor elimination, and multi‐axis rotation capabilities work together to deliver intelligent, minimally invasive surgery through the da Vinci® Surgical System.

**FIGURE 6:**
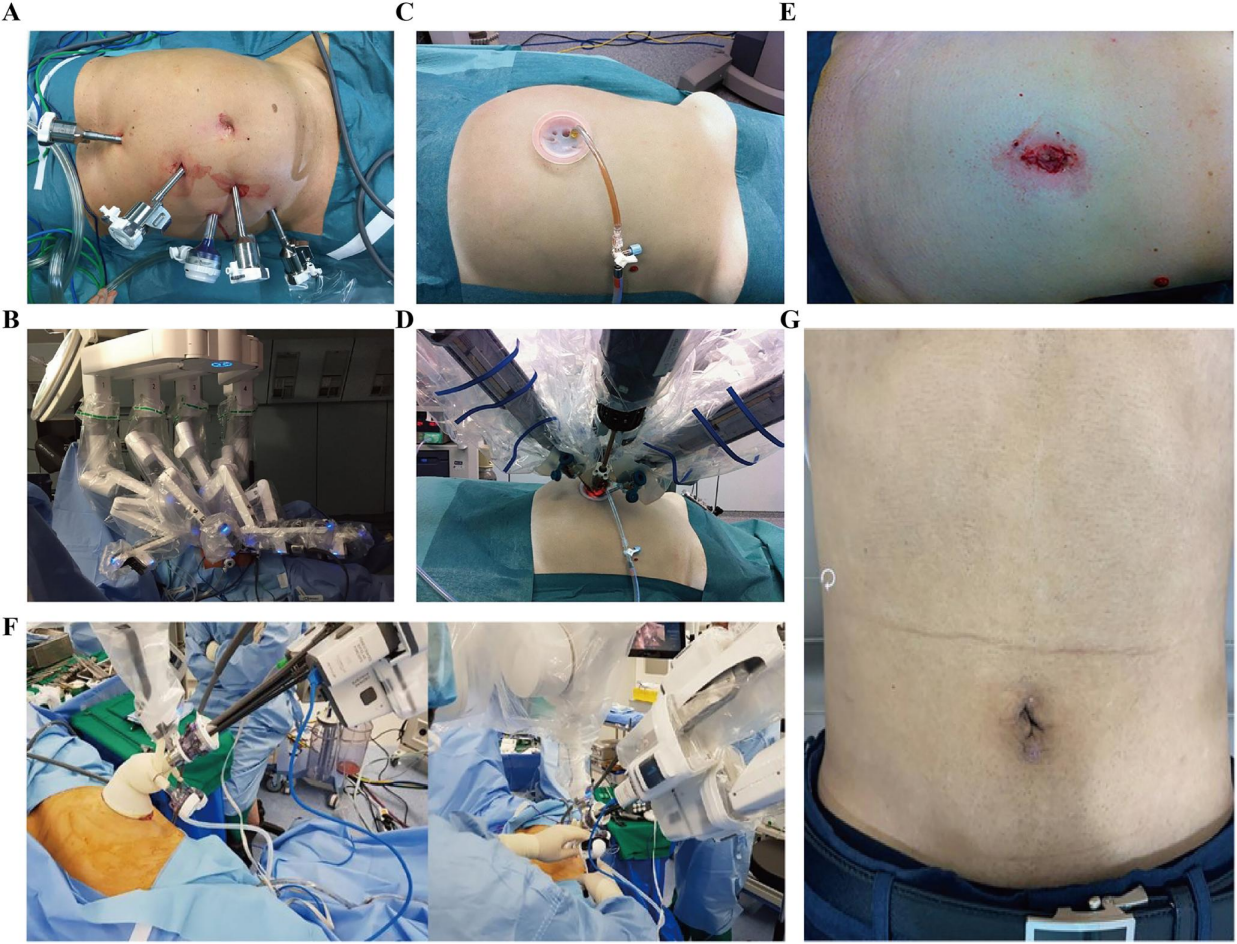
(A) Guide for da Vinci® Surgical System universal port placement. (B) Placement of the da Vinci® Surgical System in the left lateral position for surgery. Reproduced with permission.^70^ Copyright 2016, Wiley. (C) Single channel abdominal incision. (D) da Vinci® Surgical System‐to‐patient docking. (E) Image of the incision after removal of the da Vinci® Surgical System. Reproduced with permission.^71^ Copyright 2013, Wiley. (F) All robotic arms are inserted through a cannula pin. (G) Image of skin incision 2 weeks after surgery. Reproduced with permission.^72^ Copyright 2020, Wiley.

According to the improved effect for procedures in general surgery, the current use of the da Vinci® Surgical System falls into three main categories. (1) The first category is carrying out cases similar to conventional surgery in the same environment with a limited improved effect. Due to the straightforward anatomy and lack of intraoperative challenges, common procedures, like cholecystectomy, inguinal hernia repair, and appendectomy, would not demonstrate any significant advantages of the da Vinci® Surgical System. Wren et al. completed nine robotic‐assisted cholecystectomies with a mean operative time of 105.3 min, close to the 106.1 min of standard laparoscopic surgery. Also, the incidence of adverse events was the same in both groups after 3 months of follow‐up.^66^ Similarly, according to another report, da Vinci® Surgical System performs as safely and effectively as conventional surgery for inguinal hernia repair.^67^ (2) The second category is suitable for procedures that can significantly improve surgical outcomes, such as radical gastric cancer,^68^ total rectal mesenteric resection,^69^, ^70^ and colectomy.^71^, ^72^ Huang et al. compared the da Vinci® Surgical System in the perioperative period of radical colorectal cancer surgery and showed that the robotic group had a shorter operative time and less intraoperative blood loss, which facilitated postoperative recovery.^73^ In the field of radical surgery for gastric cancer, da Vinci® Surgical System also has a significant advantage over laparoscopic surgery in terms of anastomotic leak rate and mortality^74^, ^75^, ^76^ (Figure 5E,F). Similar to this, the size of the incision following a single‐ or multi‐channel surgical procedure frequently reflects the proficiency and level of the surgical robot (Figure 6). (3) Procedures that are difficult to perform laparoscopically but can be performed with robotic assistance should be regarded as the third category. Aneurysm resection often requires thorough surgical planning and experienced surgeons due to its low tolerance and high degree of difficulty. Traditional laparoscopy does not allow for difficult procedures such as abdominal aortic aneurysm resection. In contrast, the da Vinci® Surgical System is highly feasible and advantageous for assisting in abdominal aortic aneurysm resection and is now a powerful adjunctive surgical tool.^77^

**FIGURE 7:**
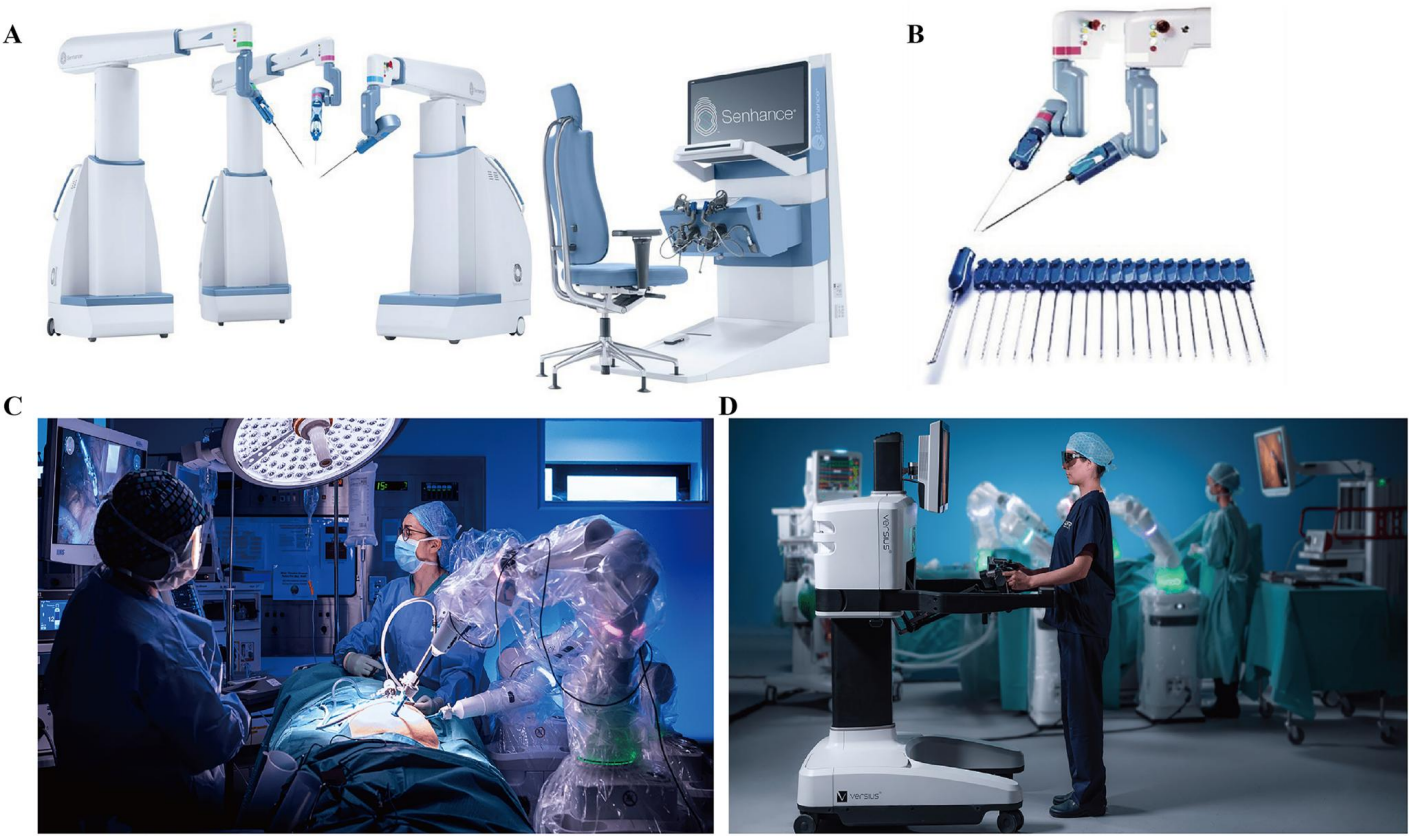
(A) Image of Senhance® robotic platform. Reproduced with permission.^79^ Copyright 2022, Asensus Surgical US. (B) Forceps system of Senhance® robotic platform. Reproduced under terms of the CC‐BY license.^80^ Copyright 2022, The Authors, published by Asia Endosurgery Task Force and Japan Society of Endoscopic Surgery and John Wiley & Sons Australia, Ltd. (C) Versius® surgical system for abdominal surgery. (D) Surgeon takes a standing position to manipulate Versius® surgical system. Reproduced with permission.^85^ Copyright 2022, CMR Surgical.

The Senhance® robotic platform (Asensus Surgical Co.), a latecomer in the field of general surgery robots since 5 years ago, uses a novel dynamic eye tracking system that focuses the camera on the field of surgeon view.^78^ The eye‐tracking technology ensures great consistency and interactivity for surgeons and helps to reduce fatigue during long procedures. Unlike the da Vinci® Surgical System, the Senhance® robotic platform has three or four independent robotic arms, each of which can move autonomously to adapt to different types of surgery^79^ (Figure 7A,B). As a representative of the open console, the surgeon can choose the most comfortable position to manipulate the arm and communicate with colleagues without any obstacles. In addition, some accessories such as forceps can be reused, which significantly reduces the cost of surgery.^80^ Hirano et al. successfully completed a double‐port sigmoid resection using the Senhance® robotic platform without any postoperative complications.^81^ Lin et al. similarly did a single‐center study on the safety and efficacy of the Senhance® robotic platform applied to colorectal surgery.^82^ The research team retrospectively analyzed 46 patients and effectively validated the feasibility of the Senhance® robotic platform in colorectal surgery.

Although da Vinci® Surgical System has been dominating the surgical robot market over the last 20 years, increasing new surgical robots are still being introduced. Some new technologies such as visual tracking, open console, or haptic feedback can be found in the most recent surgical robots on the market.^83^, ^84^ The Versius® surgical system (CMR Surgical Co.), the smallest surgical robot, is a revolutionary development in surgical robotics^85^ (Figure 7C). Another major advantage of the Versius® surgical system is its optimized human–machine interaction system.^86^ Its open console design and ergonomic seat structure give the surgeon a more comfortable operating environment (Figure 7D). Together with the high‐definition 3D camera and haptic feedback system, it improves comfort while maintaining precision. Collins et al. analyzed the data of 32 patients who completed colorectal cancer resection using the Versius® surgical system and found that all patients did not have any serious postoperative complications or deaths. For right hemicolectomy, the average operative time and blood loss were 221 min and 150 ml, which met the surgical requirements with ethical approval.^87^ Atallah et al. completed a transanal total rectal mesenteric resection with the assistance of the Versius® surgical system.^88^ A novel form of coordinated robotic and human surgery was finished by two surgeons in 195 min by performing the procedure transabdominally and transanally, convergent on the peritoneal reflex. The future surgical model will develop toward intelligence and proceduralization, and the advent of surgical robots will greatly advance this process.

### 3.4 Urological surgery robot

Surgical robots are gradually replacing traditional open surgery, but due to anatomical peculiarities, other disciplines like urology have not been able to spread as fast as orthopedics or general surgery. First of all, some routine surgeries in urology, such as nephrectomy, ureterotomy, or cystectomy for tumors, can be well adapted to surgical robots.^89^ One of them is radical prostate cancer surgery, which represents the maximum advantage of surgical robots. The surgical robot can provide the operator with a large and stereoscopic high‐definition field of view, which can present the alignment and distribution of soft tissues or organs more clearly. Not only can the tumor be finely removed during surgery, but lymph node dissection can also be performed without leaving any dead space. Some important parts such as the prostate lateral fascia can be preserved under 3D view, and some important blood vessels or nerves can be precisely anastomosed in the first place. These greatly improve the quality of surgery and minimize the impact on the physiological activity of patients.

**FIGURE 8:**
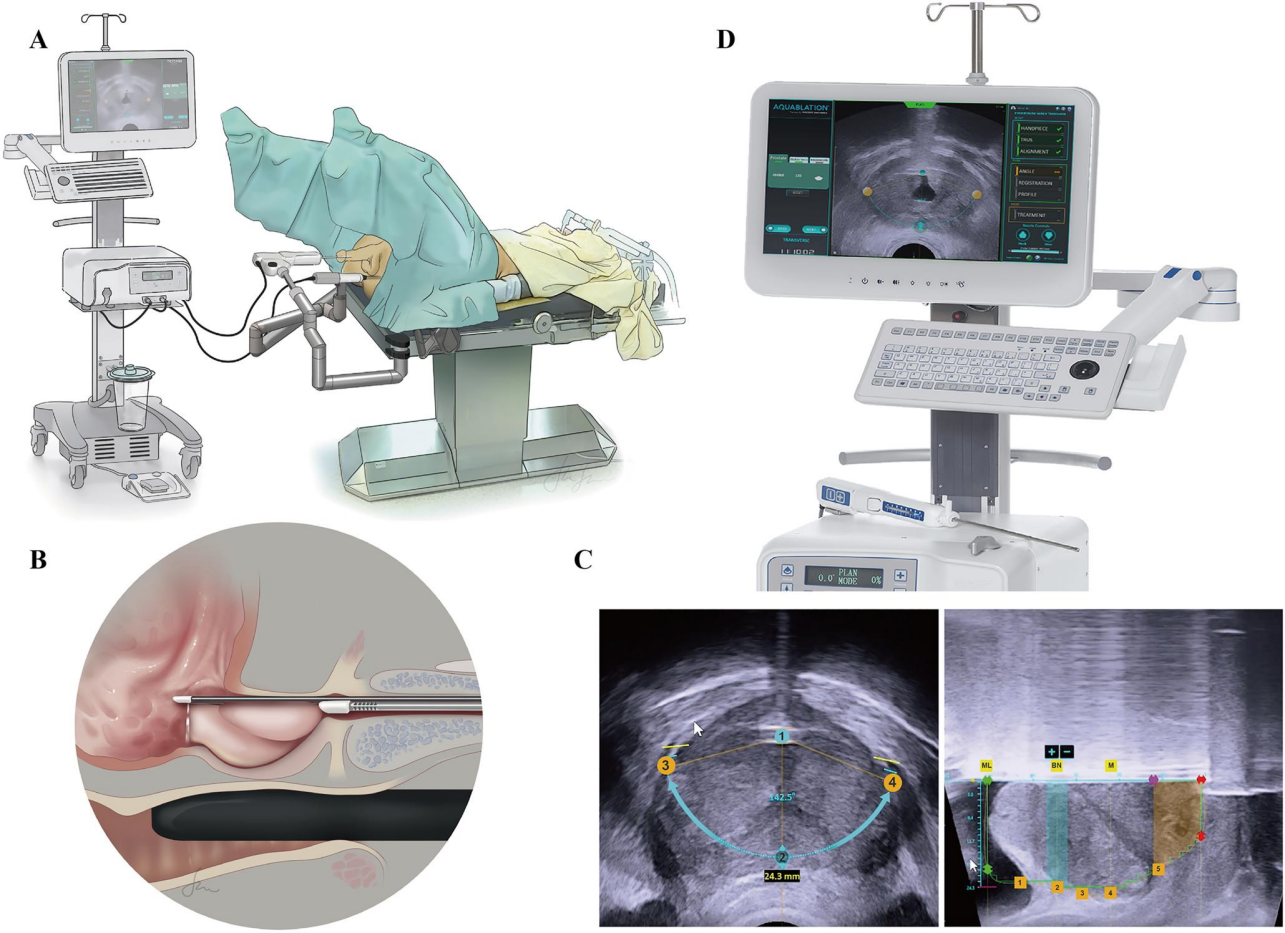
(A) Intraoperative use of the Aquabeam® system. (B) Waterjet ablation for precise and rapid removal of prostate tissue. (C) Ultrasound imaging. Reproduced with permission.^90^ Copyright 2019, Elsevier. (D) The main console of the Aquabeam® system. Reproduced with permission.^91^ Copyright 2022, PROCEPT BioRobotics Corporation.

The Aquabeam® system (PROCEPT BioRobotics Co.) innovated the introduction of aqueous ablation therapy in the treatment of urinary tract obstruction due to prostatic hypertrophy.^90^ In the face of benign prostatic hyperplasia, aqueous ablation therapy is able to visualize and preserve the anatomical structures related to sexual and urinary function and to precisely excise the diseased tissue (Figure 8A).^91^ The safety and efficacy of hydroablation therapy is comparable to that of transurethral resection of the prostate (TURP) and has a better performance in terms of improvement of postoperative complications. The Aquabeam® system is an automated surgical robot combining ultrasound image guidance and water ablation technology (Figure 8B–D). With real‐time multidimensional image judgment of the prostate, surgeons can personalize the extent of resection for each patient. Misrai et al. completed a 1‐year multicenter study to demonstrate the feasibility and safety of the Aquabeam® system in benign prostatic obstruction.^90^ Thirty patients participated in this study and none of them reported urinary incontinence or erectile dysfunction. Whiting et al. also did a similar single‐center study in the UK and showed that the Aquabeam® system was a safe and effective treatment significantly improving a range of symptoms in the low urinary tract.^92^

### 3.5 Gynecological surgery robot

Most gynecological surgeries are performed in the pelvis; therefore, the surgical field of view is usually limited. Conventional instruments are not convenient in the small space, and it is difficult to perform some fine separation or suturing operations. Traditional total hysterectomy^93^ can be done transabdominally or transvaginally. Due to the peritoneal reflex, the bladder is easily damaged intraoperatively. Also, because the ureter is adjacent to the uterus, it is advisable to clamp it close to the uterus. The more complex anatomical relationships and physiological structures force the urgent need for an auxiliary tool for gynecological surgery that can magnify the field of view and rotate in multiple angles.

**FIGURE 9:**
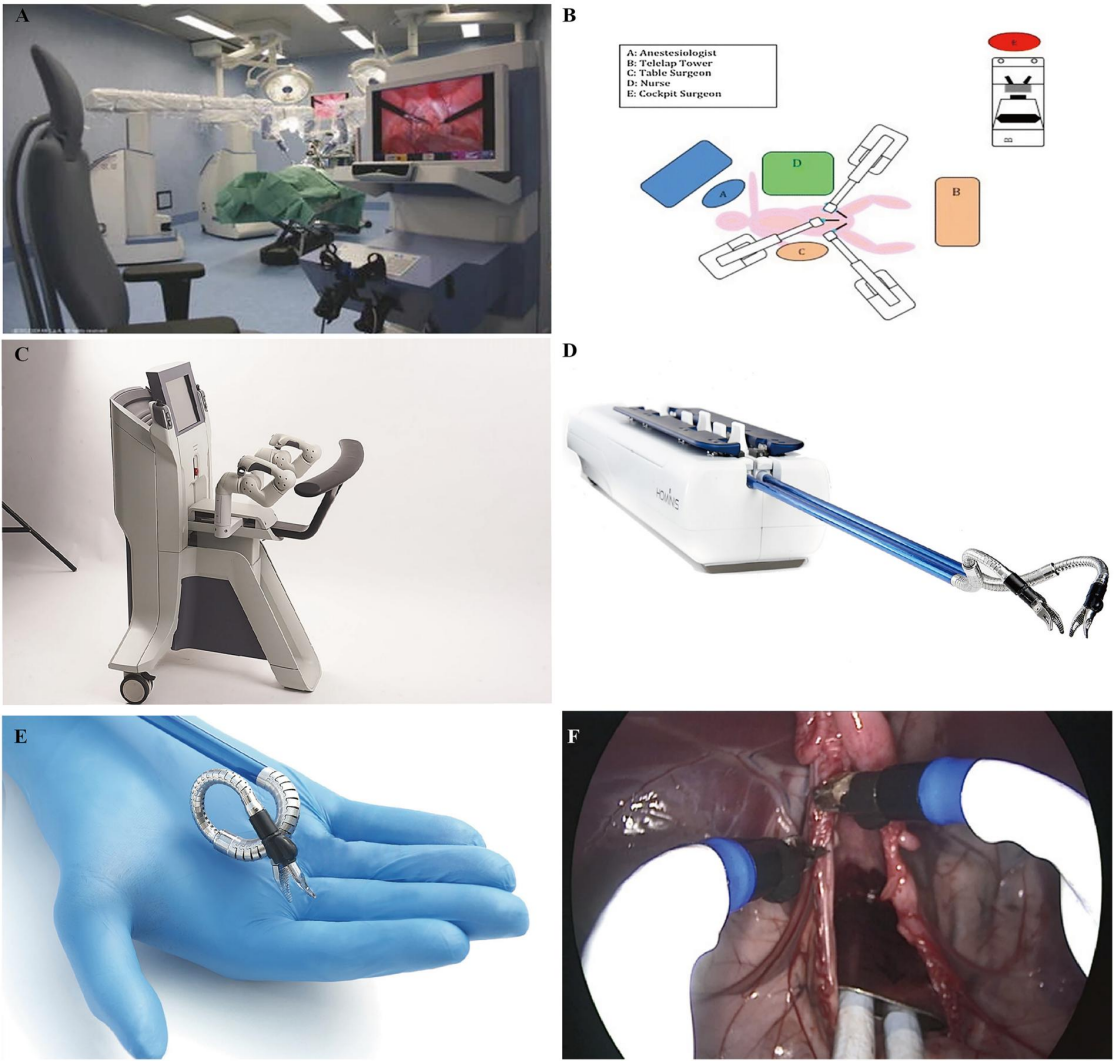
(A) Image of TELELAP ALF‐X surgical system. (B) The station in the operating room. Reproduced with permission.^94^ Copyright 2016, Springer Nature. (C) Small surgeon console. (D) Humanoid arm robot arm. (E) Multi‐plane flexible deformation. (F) Microscopic view of hysterectomy. Reproduced with permission.^98^ Copyright 2022, Momentis Innovative Surgery.

The TELELAP ALF‐X surgical system (SOFAR S. p.A. Co.) is a robot specializing in gynecological surgery and has been hailed as a new tool for gynecologists, which uses an open console with both an eye‐tracking system camera and a robotic arm^94^ (Figure 9A,B). Traditional open or laparoscopic surgery always faces different risk factors, such as massive blood loss and incomplete clearance of the retroperitoneal space. The TELELAP ALF‐X surgical system has a robotic arm that can freely move between gaps and provide real‐time feedback of high‐definition images for gynecologists. Fanfani et al. pioneered TELELAP ALF‐X surgical system‐assisted total hysterectomy + bilateral salpingo‐oophorectomy + pelvic lymph node dissection.^95^ Eighty women received robotic surgery and 93.7% of them successfully completed the procedure without conversion to laparoscopic or open surgery. The feasibility and safety of the TELELAP ALF‐X surgical system for total hysterectomy was firstly validated. In addition, Fanfani et al. also reported the outstanding performance of the TELELAP ALF‐X surgical system in the treatment of early endometrial cancer.^96^

The Anovo™ Surgical System (Momentis Innovative Surgery), the only FDA‐authorized vaginal surgical robot, showed the longest reach into the body (Figure 9C). The multi‐joint feature gives it a high level and multi‐planar flexibility (Figure 9D,E). The 360° rotation capability can provide excellent obstacle avoidance ability and operating angle. Pelvic organs, such as the uterus, fallopian tubes, or ovaries, are deeply located and have complex surrounding structures; therefore, traditional lumpectomy often results in heavier pain and large scars.^97^ To further reduce the chance of infection and recovery time, minimizing the incision is the greatest goal of surgery via the natural cavity. A recent clinical study showed that the Anovo™ Surgical System was fully capable of performing hysterectomy (Figure 9F).^98^ In addition, it has a great potential for tubectomy, oophorectomy, or cystectomy.

### 3.6 Neurosurgical robot

The successful use of surgical robots in the field of neurosurgery has also gained lots of acceptance. In neurosurgery, anatomical restrictions and poor visualization are also mutual problems.^7^ Procedures such as common intracranial hemorrhage drainage^99^ and debridement decompression^100^ require preoperative assessment of incision size and access as well as intraoperative vigilance for intracranial nerve and vascular injury. All neurosurgical procedures should be performed to achieve adequate visualization while controlling bleeding and preserving neurovascular function to the maximum extent possible to complete resection or decompression. The strong performance of the robot relative to traditional craniotomy gives a good foundation for future clinical popularity.

**FIGURE 10:**
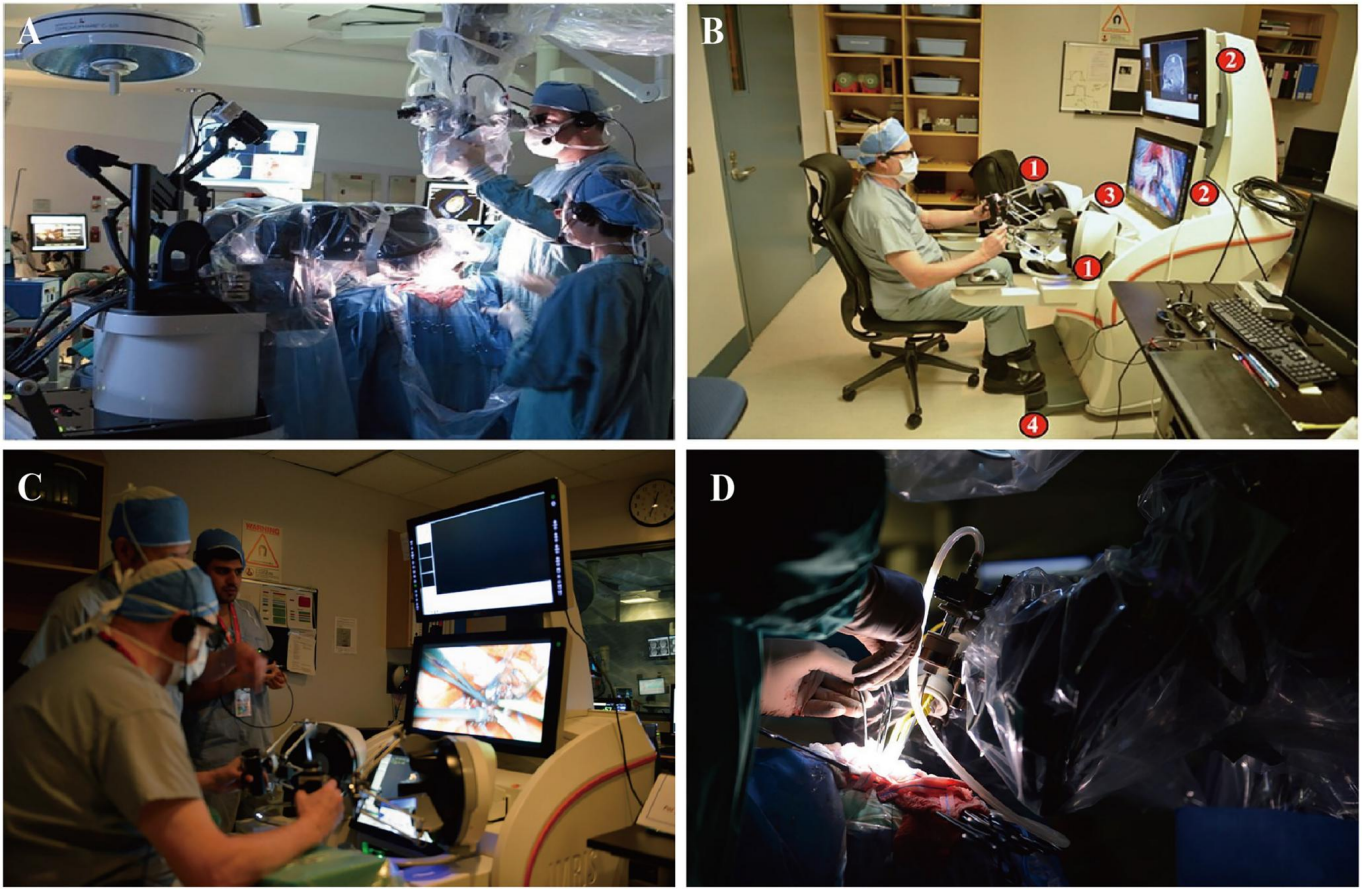
(A) The neuroArm located inside the operating room in the Foothills Hospital. (B) The surgeon operates the neuroArm controller. (1) haptic devices, (2) displays, (3) touch screen display, and (4) foot pedal. Reproduced under terms of the CC‐BY license.^104^ Copyright 2016, The Authors, published by Hindawi Publishing Corporation. (C, D) The working scene of the neuroArm. Reproduced with permission.^103^ Copyright 2022, NeuroArm‐UCalgary.

The NeuroArm (IMRIS‐deerfield Co.) is the first MRI‐compatible image‐guided neurosurgical robot.^101^ The NeuroArm has the ability to operate within a high magnetic field, and the surgical team will not be interrupted by intraoperative imaging.^102^ The improved robotic arm controller helps surgeons maneuver better while providing timely haptic feedback (Figure 10A,B). In addition, the surgical assistant can perform surgery with the NeuroArm while all image data are shared to ensure maximum consistency and accuracy^103^ (Figure 10C,D). The NeuroArm is the robot of choice for neurosurgery because of its intraoperative “see‐through” capability, incorporating MRI imaging technology. The team compared the mechanical signals between the forceps tips and the brain tissue at different gradations and found that the differences in mean values could be related to the position of the robotic arm, the depth and size of the tumor, and the subtype of the tumor.^104^ Recorded force data may be of value for surgical education and case rehearsal and can contribute to the development of neurosurgical simulators whereby information of tool–tissue interaction forces will allow modeling of the brain tissue in health and disease.

### 3.7 Cardiothoracic surgery robot

Cardiothoracic surgery, a high‐risk and invasive procedure, needs long postoperative recovery time and high mortality rate.^105^ The introduction of surgical robots will remarkably benefit for a greater improvement in the quality of surgery.^106^ Procedures, such as lobectomy or valve transplantation, often require the establishment of extracorporeal circulation and separation of the rib cage.^107^, ^108^ In addition to disfigure the appearance of patients, excessive thoracic surgical incisions also raise the danger of infection and delayed healing. The use of robotic surgery for lobectomies is currently safe and practicable.^109^ The cost of the robotic platform will gradually drop with the rising number of procedure cases and postoperative complications will also be increasingly rare. For valve replacement, which is common in cardiac surgery, robotic aortic valve replacement through a small incision in the lateral chest wall allows for early extubation with minimal invasiveness and without the need for postoperative analgesics.^110^ There are no surgical robots dedicated to cardiothoracic surgery, and both the da Vinci® Surgical System and the Versius® surgical system are approved for cardiothoracic surgery.

## 4 SUMMARY

Over time, surgical robots have been increasingly accepted by clinicians and patients. Surgical robots could assist surgeons effectively in minimally invasive surgery through their flexible bionic robotic arms, complemented by 3D high‐definition images. The multi‐planar rotating robotic arm is the core component of the surgical robots to complete operations, such as suturing and hemostasis. Common arm designs include single‐channel arms, multi‐channel arms, and flexible arms. Different robotic arms can be adapted to different cavities and anatomical structures to perform some operations with precise force. What closely coupled with the robotic arm is the image presentation system. It has a 3D magnification effect that further enhances the surgeon's ability to discern the surgical field. Some of them are even equipped with an eye tracking system to maximize the smoothness of human–machine cooperation. As one of the three major components of a surgical robot, the main console is the closest part linked with the surgeon. Traditional split consoles require the surgeon to operate from a distance. The ergonomic seat reduces physical fatigue from long surgeries. The new open system of console gives the surgeon more flexibility and comfort in the operating space. This facilitates smooth communication between surgeons and assistants standing by patients. The rational and scientific design of the surgical robot is a prerequisite for good rehabilitation results.

Each surgical robot has a range of optimal use in the face of the different needs of each clinical subspecialty. Orthopedic surgery requires a high degree of precision in the positioning of implants. A biomechanical force line remodeling process is an important guarantee of a good surgical outcome for patients. Common orthopedic robots, such as the TSolution‐One®, not only perform implant placement but also automate osteotomies. The preoperative imaging data can be calculated to determine the ideal osteotomy volume, reducing complications brought on by force line mismatch. Additionally, intraoperative automatic irrigation lessens the thermal consequences of border friction. Besides, a range of general surgery robots, represented by the da Vinci® Surgical System, have been active in many common procedures in the gastrointestinal tract for long. Neither traditional open surgery nor the emerging laparoscopic surgery can shock the core superiority of surgical robots. Robots, such as the da Vinci® Surgical System, offer 3D imaging and a wide range of surgical applicability. Advanced tremor filtering and fewer personnel requirements have also made the da Vinci® Surgical System the most popular surgical robot in the world. Surgical robots also showed rapid growth in areas with deeper anatomy, such as gynecology, neurosurgery, and urology. Neurosurgical robots have integrated impact localization capabilities that reduce radiation while indirectly reducing operative time. Gynecology and urology are often dominated by noninvasive procedures in which the natural cavity is preferred. The slender snake‐like robotic arm can be adapted to different cavities for specific biopsy or resection tasks.

The material selections, rotation angles, and overall dimensions of the robotic arms are particularly important. In addition, in the complex abdominal cavity environment, the surgical robot needs to maintain high pixel and precision under the condition of full of body fluid and blood. Some corresponding functions, such as carbon dioxide release and electrocoagulation, also need to be coordinated with the robotic arm for accuracy. If the front side of the robot arm equips sensors or chips, it can help it to independently identify different situations and complete corresponding operations, which will greatly increase the safety and intelligence of surgical robots. At the same time, the design of the robotic arm should be personalized for different parts of the body or different sizes of tissues. It enters the body minimally and invasively through different accesses, avoids important anatomical locations, and completes specific resection or clamping. In the complex and narrow surgical environment of the future, the accuracy and flexibility of the robotic arm will be the main development direction.

## 5 OUTLOOK AND CHALLENGES

**FIGURE 11:**
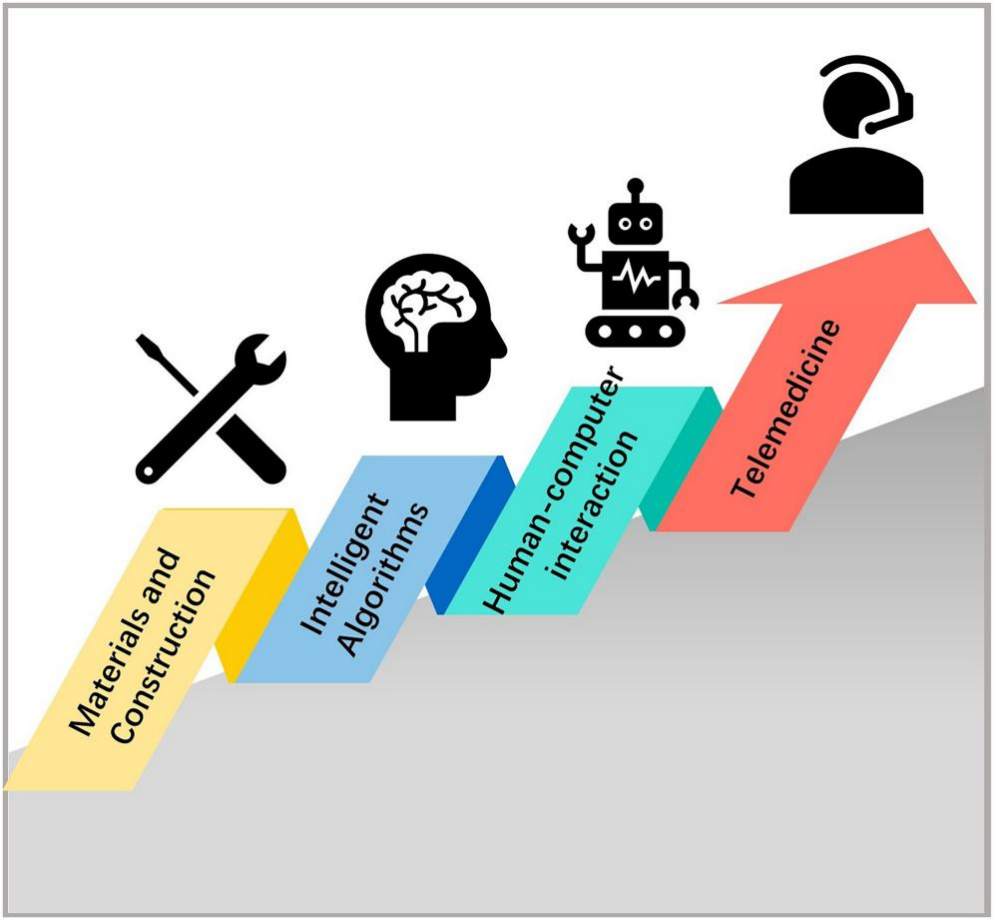
The future direction of surgical robots.

After decades of promotion in different disciplines and fields, surgical robots start to become competent in many medical scenarios. With the popularity of minimally invasive surgery in various clinical fields, the development of surgical robots also comes into a golden era. The core advantages of surgical robots gradually draw the attention of physicians, hospitals, and patients, which include smaller incisions, less bleeding, and shorter recovery times. However, the widespread popularity and even large expansion of surgical robots today also revealed many drawbacks. The first issue is the excessive cost of purchase and maintenance. Not all hospitals can afford to introduce the da Vinci® Surgical System because it can cost up to RMB 20 million. Hospitals and patients are under a great deal of financial strain due to its significant costs for routine maintenance and replacement of consumables. The high price of surgical robots may be due to limited production and technological monopoly. Besides, the flexibility of the robot arm, the intelligence of the pre‐programmed procedures and the functionality of the materials all influence the price of surgical robots. If manufacturers could focus on particular modules separately, costs would significantly be reduced. Second, the large size and enormous weight of the robotic arms and other components make it impossible to move freely in the operating room. The cumbersome feature of the instruments also challenge surgeons to plan well before surgery. The capability to follow up on the disposal is also necessary, in case it perishes or suffers damage mid‐procedure. The safety and smoothness of the procedure could be well ensured by having a technician on site or remotely online to guide the machine during the initial stage of use. At the same time, someone needs to be dedicated to moving, debugging, and maintaining the daily operation of the machine in the operating room. This not only extends the service life but also prevents accidental damage to the machine. Finally, the imperfect haptic feedback function continues to plague the majority of surgeons. The inability to discern with accuracy on how difficult to make a knot, how frequently to cut, or how much tissue to tear. This greatly increases the nonessential error rate of surgeons and also prolongs the learning cycle with the machine. Whether the algorithm could be optimized to achieve better human–computer interaction is the first task to solve such problems. In the future, we should integrate cutting‐edge technologies and pay attention to the development trends and improvement directions of surgical robots. There are still some issues worth exploring (Figure 11):

1. Surgical robots should continue to develop for miniaturization and lightness. The majority of current surgical robots have a tendency to be heavy, large, and less flexible when moving around the operating room. Smaller surgical robots, such as the Versius® surgical system, are increasingly favored by hospitals. Each arm has a separate trolley that can be freely adjusted in angle and orientation while greatly reducing the footprint. In addition to the necessary metal parts, haptic or flexible materials should be used more often in the construction of the main console and the robotic arm. The performance and functionality of surgical robots can be enhanced by new materials, enhancing the surgical process safety, adaptability, and precision. Miniaturization will no longer be the only design objective in the future, advanced and lightweight functional materials should also be considered when selecting materials.
2. Intelligent algorithms should gradually become another core of surgical robots. The correct presetting of the program is required for the robot arm gripping, moving, and telescoping functions. To face different types of surgery and different operating habits of surgeons, intelligent surgical robots should adjust the preset procedures in time to meet different surgical needs. At present, robots based on existing algorithms cannot fully match the requirements of high safety, flexibility, precision, and low latency. If advanced technologies, such as artificial intelligence, big data computing, and cloud‐based storage, are combined, the robot's cognitive, decision‐making and execution systems will be well enhanced. Due to the specificity of clinical medicine, it is difficult for surgical robots to achieve the goal of a high level of human–computer interaction. The main console should have high stability and high feedback at least, since it is the component that interacts with the surgeon in the closest way. Whether the main console can successfully complete the corresponding operation with the surgeon according to the established plan is an important assessment indicator for the robot to have good interactivity. On the contrary, the surgical robot should also take the initiative to identify the surrounding and intraoperative conditions, to achieve real‐time monitoring and warning, and to help surgeons avoid mistakes. Both humans and machines should be capable of tracking and sensing in this dynamic coordination.
3. A wider range of adaptability is an important indicator to examine for promoting surgical robots. Nowadays, the common surgical robots available on the market mostly focus on general surgery, orthopedics, and gynecology. This is because the surgical procedures are simple, with clear anatomical structures and fixed patterns. However, these surgical robots do not work well in other areas of medicine such as dermatology, cardiology, or respiratory medicine. Dermatology mostly operates on body surface swellings rather than cutting through the natural cavities of the body. It is hoped that a series of noninvasive surgical robots will be developed in the future with electrocoagulation, radiofrequency ablation, or ultrasound as the basic means.
4. With the advent of the 5G era, the powerful combination of virtual reality and network communication technology has created a new way of remote surgery. In the future, doctors will be able to complete surgeries based on live images, regardless of location or number of people. The doctor actions at a distance will be converted into digital signals and transmitted to the surgical robot in front of the patient. The surgical robot is controlled remotely, which requires a high level of network transmission. At the same time, the surgical robot itself needs to be equipped with the appropriate modules for receiving and converting signals. This will greatly increase the ability to release medical resources and globalize surgery.

## AUTHOR CONTRIBUTION

*Conceptualization*: Wei Chen. *Data curation*: Chao Li, Tongtong Zhang, and Haoran Wang. *Investigation*: Zhiyong Hou and Chao Li. *Methodology*: Chao Li, Haoran Wang, and Yingze Zhang. *Supervision*: Wei Chen and Yingze Zhang. *Validation*: Wei Chen. *Writing – original draft*: Chao Li, Tongtong Zhang, and Haoran Wang. *Writing – review & editing*: Zhiyong Hou, Yingze Zhang, and Wei Chen.

## CONFLICT OF INTEREST

The authors declare no conflict of interest.
